# Promoting psychological well-being in preschool children: study protocol for a randomized controlled trial of a mindfulness- and yoga-based socio-emotional learning intervention

**DOI:** 10.1186/s13063-022-06979-2

**Published:** 2022-12-27

**Authors:** O. Courbet, Q. Daviot, V. Kalamarides, M. Habib, M-C C Castillo, T. Villemonteix

**Affiliations:** 1 Laboratory of Psychopathology and Processes of Change [LPPC], Paris-Lumières University, Saint-Denis, France; 2 Abdul Latif Jameel Poverty Action Lab [J-PAL], Paris School of Economics, Paris, France; 3 DysCo Laboratory, Paris-Lumières University, Nanterre, France

**Keywords:** Psychological well-being, Mindfulness, Yoga, Kindness, Socio-emotional learning, Socio-emotional competencies, Self-management, Self-regulation, Connection

## Abstract

**Background:**

Mental health issues in youths have cascading negative effects on school outcomes, professional life, and physical health. Psychological well-being (P-WB) is an important protective factor against mental illness. Preliminary research suggests that mindfulness- and yoga-based socio-emotional learning (SEL) interventions can each have a positive impact on preschoolers P-WB. The objective of this trial is to rigorously evaluate the effect of a 24-week combined mindfulness- and yoga- based SEL intervention in preschool children from a French socio-economically disadvantaged area.

**Methods:**

The P-WB promotion intervention is compared to a wait-list control condition in a cluster randomized controlled trial (RCT). Sixty-four pre-Kindergarten classrooms are randomized to the intervention or control group. Primary outcomes measure self-management capacity and core P-WB components: connection, insight, engagement, and positive relationship. Secondary outcomes include measures of mental health, executive functioning, and school performance. Primary and secondary outcomes are assessed through teacher questionnaires, standardized observations of children in school context, and experimental tasks and by collecting results of the national evaluation at first grade. All children-level outcomes are evaluated at pre-intervention, at the end of the intervention, and 1 year later (follow-up analysis), to the exception of school performance which is evaluated at follow-up only. Intention-to-treat analyses, accounting for clustering within classes, will adopt a random effects linear regression model to examine outcomes for the intervention versus control children.

**Discussion:**

This is the first trial to rigorously evaluate a combined mindfulness- and yoga-based P-WB promotion intervention, and the first RCT evaluating a SEL curriculum in French schools. Results may have key implications for P-WB promotion in preschool children.

**Trial registration:**

https://www.drks.de/ DRKS00028623. Retrospectively registered on 30 May 2022

**Supplementary Information:**

The online version contains supplementary material available at 10.1186/s13063-022-06979-2.

## Administrative information

Note: the numbers in curly brackets in this protocol refer to SPIRIT checklist item numbers. The order of the items has been modified to group similar items (see http://www.equator-network.org/reporting-guidelines/spirit-2013-statement-defining-standard-protocol-items-for-clinical-trials/).

**Table:** 

Title {1}	Promoting psychological well-being in preschool children: study protocol for a randomized controlled trial of a mindfulness- and yoga- based socio-emotional learning intervention
Trial registration {2a and 2b}.	https://www.drks.de/ Number: DRKS00028623. Registered on 2022/05/30, retrospectively registered.
Protocol version {3}	September 2021, third version.
Funding {4}	This trial received public funding from the regional health agency (*Agence régionale de Santé*) of the *Seine-Saint-Denis* French department, and private funding from the local foundation of Paris Airports (*Fondation des aéroports de Paris*).
Author details {5a}	Courbet^a^, O., Daviot, Q.^b^, Kalamarides, V.^a^, Habib, M.^c^, Castillo, M.C. ^a^, Villemonteix, T.^a^ ^a^ Laboratory of Psychopathology and Processes of Change, Paris-Lumières University ^b^ Abdul Latif Jameel Poverty Action Lab, Paris School of Economics ^c^ DysCo Laboratory, Paris-Lumières University
Name and contact information for the trial sponsor {5b}	Thomas Villemonteix thomas.villemonteix@univ-paris8.fr
Role of sponsor {5c}	The sponsor of this trial is Paris-Lumières University. In particular, members of the LPPC collect, analyze and interpret data, in collaboration with members of two other laboratories (Abdul Latif Jameel Poverty Action Lab [J-PAL], Paris School of Economics and DysCo Laboratory, Paris-Lumières University).

## Introduction

### Background and rationale {6a}

Mental health issues are common in children and adolescents: psychiatric disorders affect at least one in ten 5-to-16-year-old youths [19], and 50% of all lifetime mental illness begins by age 14 [49]. Emotional and behavioral problems that are not part of a diagnosed disorder are even more common, and their rates have increased over the past 30 years in several countries [19], with a recent dramatic peak due to the COVID-19 pandemic [57, 63]. Mental health issues in youths can have cascading effects over time on school and professional outcomes, contributing to a major economic burden [33]. In this context, reinforcing public action to promote mental health in youths has recently been described by the American Academy of Pediatry as a “national emergency” [4].

Psychological well-being (P-WB) is a multidimensional construct whose definition remains a matter of debate [31]. According to the PERMA framework, P-WB in youths can be measured based on five core facets of self-evaluation: *Positive Emotions*, the tendency to experience hedonic feelings of happiness; *Engagement*, the psychological connection to activities or institutions; *Positive Relationships*, the feeling of being socially integrated, cared about and supported by others; *Meaning,* the belief that one’s life is valuable; *Accomplishment*, the feeling of being capable of making progress and of achievement [48]. Decades of observational and interventional research have linked measures of youths and adults’ P-WB to health outcomes, as well as to educational and professional achievement [18, 30, 82]. While measures of P-WB are partly independent from measures of mental illness, research suggests that psychological well-being is an important protective factor for mental disorders [45, 50, 70, 85].

Gathering children from various backgrounds for a substantial daytime, school context constitutes a preferential context for P-WB promotion through socio-emotional learning (SEL [4];). At the individual level, a recent model identified 4 skills central to P-WB (P-WB skills): *Awareness*, defined as an heightened and flexible attention to perceptual impressions and internal cues such as thoughts or emotions; *Connection*, a subjective sense of care and kinship toward other people; *Insight*, a self-knowledge concerning the manner in which emotions, thoughts, and beliefs contribute to one’s subjective experience; *Purpose*, a sense of clarity concerning personally meaningful aims and values [24]. Beyond these four specific skills, it is probable that other related individual competencies are important for children’s P-WB. Socio-emotional competencies (SEC) designate the set of social and emotional abilities necessary to function effectively in social context and deal efficiently with the social and emotional challenges of everyday life [17]. The Collaborative for Academic, Social and Emotional Learning (CASEL) framework, one of the dominant SEC frameworks, identifies five core SEC. Among these, *Self-management*, the ability to regulate one’s emotions, stress, impulse, thoughts, and behaviors, has been identified as a key predictor of school engagement [72] and school achievement [51, 78], suggesting a strong impact on the P-WB *Engagement* dimension. Given the key relationship between *Self-management* and school readiness, SEL interventions targeting P-WB skills and *Self-management* in preschool years may have unique developmental leverage [10].

Among the various approaches available to promote P-WB, mindfulness-based interventions (MBIs) have demonstrated the greatest efficacy in both clinical and non-clinical populations [79]. Mindfulness describes both a mental faculty (a “trait”) and a practice implying deliberate conscious awareness of the present moment, including meta-cognitive awareness of present thoughts or emotions without judgment [37, 69]. Directly targeting the *Awareness* component of P-WB skills, mindfulness practice is also thought to contribute indirectly to the three other core P-WB skills—that is *Connection, Insight*, and *Purpose* [76]. Moreover, constructs measured to evaluate MBIs in a school context overlap conceptually with core competencies of the CASEL SEC framework—especially *Self-management*, and MBIs have been found to enhance *Self-management* capacity [36]. Along MBIs, yoga-based interventions (YBIs) have been identified as promising P-WB promoters. Focused on controlled breathing, body movements, and postures, yoga shares with mindfulness practice a direct focus on *Awareness*, while also providing a space to develop *Self-management* skills [76]. In youths, MBIs have been found to promote executive functioning and attention (two key contributors to *Self-management* competency) and to reduce depression, anxiety/stress, and negative behaviors [35], while YBIs were found to reduce anxiety [80]. Effects of yoga breathing practices are reflected in changes in functional brain connectivity and changes in the activity of brain regions involved in emotion processing [59]. In preschoolers, preliminary studies suggest that mindfulness and yoga practice positively impact several components of executive functioning (visual attention, sustained attention, inhibition), *Self-management* capacity, and pro-social behavior, while diminishing externalizing symptoms [64, 73].

In sum, a strong evidence-base for MBIs exists in the overall P-WB promotion field, along with promising data for YBIs. Nonetheless, recent literature reviews have underlined that available studies of MBIs in school-aged children are characterized by important methodological limitations [37, 67]. Most studies were conducted in small samples, few integrated independent blind observer ratings, and studies were each devoted to testing new mindfulness-based protocols rather than replicating previous findings [67]. In preschoolers more specifically, the current evidence-base for MBIs and YBIs can only be considered preliminary, as the limited number of studies available present a methodological risk of bias, with a majority presenting a high level of risk [73].

MBIs and YBIs share common targets [76], and yoga practice has been found to promote a mindfulness state [68], suggesting that combining mindfulness and yoga practice may have a synergistic impact. While two studies from a research group examined the effect of mindful yoga in preschoolers ((Razza et al. [65, 66]), to our knowledge, no study to date evaluated the effect of an intervention combining separate yoga-based and mindfulness-based activities. Furthermore, previous studies examining MBIs and YBIs often evaluated interventions delivered by specialized instructors [73]. While having external trainers delivering programs may maximize intervention quality, it can represent a major obstacle to countrywide systemic dissemination. Teacher training represents a less costly alternative and a facilitator for dissemination, while integration of mindfulness- and yoga-based activities into teacher curriculum may have a positive impact on teacher-student relationships [11].

Considering the need to improve the evidence-base for early P-WB promotion in preschoolers through replication studies with minimal risk of methodological bias, the present study was designed to rigorously evaluate a mindfulness- and yoga-based SEL curriculum delivered by trained teachers in preschools in France. While PISA studies have repeatedly found delays in SECs in French students [2], evidence-based SEL programs adapted to the national context are currently lacking. The mindfulness-based SEL protocol was adapted to the national context based on a program targeting the *Awareness*, *Connection*, and *Insight* P-WB skills, which was found to positively impact social competence and engagement in learning in a previous randomized controlled trial (RCT) [38], and delivered as part of a broader SEL curriculum integrating yoga-based activities and an emotion circle time targeting the *Insight* and *Connection* components of P-WB. Given the strong association between socio-economic status and self-management [58] or P-WB [62], we chose to deploy and evaluate this intervention in a predominantly socio-economically disadvantaged French department.

## Objectives {7}

This trial evaluates the impact of an incremental P-WB curriculum delivered in French preschools, (1) after 24 weeks of program exposure in children 4 to 5 years old (during *moyenne section* in France: US Pre-K equivalent) and (2) 1 year later (end of *grande section:* US kindergarten equivalent; follow-up analysis).

Our primary objective is to assess the effects of the curriculum on P-WB-related measures of *Connection, Insight*, *Engagement*, *Positive Relationships*, and *Self-management*. Our secondary objective is to document the effects of the curriculum on measures of mental health, executive functioning, and school performance [51]. We hypothesize that the intervention will lead to more favorable outcomes on P-WB, mental health, and executive functioning measures after 24 weeks of program exposure, that these effects will be maintained 1 year later, and that school performance at the national evaluation 2 years later will be superior in children who received the intervention compared to the control group.

We also investigate heterogeneous effects according to teacher-level and children-level characteristics. In terms of teacher-level characteristics, we first investigate heterogeneity according to teacher P-WB since higher levels of P-WB are associated with higher impacts in various interventional settings [25, 47, 52, 75]. Second, we will use a machine-learning model to assess potential heterogeneous effects of the curriculum according to the teachers’ level of commitment in implementing the intervention (see the “Methods for additional analyses (e.g., subgroup analyses) {20b}” section for additional details). Higher levels of fidelity of implementation are associated with higher gains in children’s P-WB, mental health, and executive functioning [40, 56]. In terms of children-level characteristics, we investigate heterogeneity according to the initial levels of EF, *connection*, and problem behaviors. Lower pre-intervention levels of EF and *connection* are associated with higher gains in EF [38, 73] and *connection* [73] respectively. Similarly, higher pre-intervention levels of problem behaviors are associated with higher reductions in post-intervention problem behaviors [38].

## Trial design {8}

The trial described in this protocol is a superiority two-armed cluster randomized controlled trial designed to evaluate the value-added of a P-WB promotion curriculum for pre-K children delivered by teachers, compared to teaching as-usual. Sixty-four pre-K classrooms from 50 different schools are randomly allocated to the intervention group or to the control group. In France, preschool classes can either include only students from one school year (here, Pre-K students only, “*moyenne section*” corresponding to 4-year-old children on average) or a mixture of children from Pre-K and kindergarten levels (kindergarten, hereafter K, students, or “*grande section*”, correspond to 5-year-old children on average). In the present sample, 36 classes included only Pre-K students, and 28 classes included a mixture of Pre-K and K levels students. In this context, as we expected potential differential effects according to the type of classroom, we stratified the sample by classroom type in order to compare treatment and control groups within the same type of classrooms. In practice, in the 36 classrooms with only Pre-K students, we randomly assigned 18 classrooms to the treatment group and 18 classrooms to the control group. In the 28 classrooms with a mix of Pre-K and K students, we assigned 14 classrooms to the treatment group and 14 classrooms to the control group. Overall, the treatment group and the control group are both composed of 32 classrooms.

## Methods: participants, interventions, and outcomes

### Study setting {9}

The study was conducted in public schools from sixteen municipalities of the *Seine-Saint-Denis* French department (93) (*Ile-De-France* region, France). The 93 department has the highest poverty rate of metropolitan France, placing this trial in a relatively high poverty context.

### Eligibility criteria {10}

*Inclusion criteria*: children at pre-K level (*moyenne section*) attending public schools.

*Exclusion criteria*: parent refusal for the child to participate in the study or consent withdrawal during the study.

*Specific exclusion criteria for collection of experimental data and observations*: (1) children showing comprehension difficulties in French language; (2) children with high difficulties in expressive French language or children who do not speak French; (3) children with suspicion of neurodevelopmental disorders (notably intellectual disability or Autistic Spectrum Disorder); (4) children with severe behavior problems (e.g., high aggression/tantrum level) whose teachers judged that taking part in the experimental part of the protocol would not be possible. These criteria were implemented to ensure that experimental data collection would be feasible with the selected children. Teachers were asked to exclude children based on this list of criteria.

### Who will take informed consent? {26a}

Informed consent is obtained from children’s parents, before starting the first evaluation session. Information letters with an attached reply form are posted to teachers at the beginning of the school year. Teachers then transmit these letters to parents. Parents who refuse participation for their child send the reply form back to the teacher, who then informs principal investigators of parent refusal [OC, TV]. In the information letter, (1) objectives, contents, and attended benefits of the intervention (if applicable) are described; (2) parents are told that participation of their child is entirely voluntary (i.e., parents can accept or refuse to participate to the study without any consequences), and (3) that they can withdraw their participation at any time by stating it to the teacher and/or returning the refusal reply form. Parents are not paid for their child participation in the study. Oral consent was obtained from children before experimental task data collection.

### Additional consent provisions for collection and use of participant data and biological specimens {26b}

N/A. No biological specimens are collected in this trial.

### Interventions

#### Explanation for the choice of comparators {6b}

Pre-K and kindergarten French programs do not include coherent SEL components targeting P-WB. Nonetheless, teachers sometimes decide to integrate some SEL activities on an autonomous basis. In this trial, children from classes receiving our structured and progressive P-WB promotion curriculum as part of their school year program are compared to children from waiting-list classes exposed to a “usual” school year. Teachers are randomly assigned to the intervention group or to the waiting-list control group. Teachers from the control group are told to teach their class as they would have any other year. Teachers allocated to the wait-list control group for the evaluation year (September 2021–June 2022) are proposed to follow the program training course the following year (September 2022–June 2023). Comparison to a wait-list control condition was chosen in this study to replicate intervention benefits, prior to studying the specificity of effect in future studies by comparing the P-WB promotion curriculum to active conditions. Allowing teachers to benefit from the program and materials 1 year later was deemed necessary to maximize recruitment chances in the context of the 93 French department and minimize the risk of control group disengagement from the study.

#### Intervention description {11a}

##### Wait-list control group

Teachers allocated to the control group continue to carry on their normal academic activities. Normal academic activities in French public preschool include basic language and literacy skills development, visuo-motor skills development through physical activities, artistic activities, basic numeracy skills development, and exploration of the living world, matter, and objects. P-WB promotion is not targeted in this program, although (1) physical or artistic activities may indirectly target P-WB and (2) some teachers may decide to include some activities devoted to P-WB or SEC promotion on an autonomous basis (data is collected at the end of the school year to monitor these two possibilities in the control group).

##### Intervention group

The P-WB promotion intervention is composed of a set of activities delivered each week: (1) a mindfulness-based SEL curriculum, (2) ritualized yoga activities adapted for preschool children, and (3) a ritualized circle time. Teachers in the intervention group are asked to implement the P-WB curriculum during regular school hours after completion of the training course. They receive a 2-day training delivered by the principal investigator [TV, clinical psychologist trained in cognitive-behavioral therapy including mindfulness], a yoga-instructor, and a teacher with experience in delivering yoga-based activities to kindergarten classes. Training is based on role-play to directly experience teaching of P-WB activities and includes a personal initiation to mindfulness and yoga. Curriculum is set up to be delivered during 24 weeks. A guided instruction manual, with detailed descriptions of activities, objectives, timing, and contents is provided for each type of activity (kindness curriculum, yoga, circle time) along with all required material.

1. *French adaptation of the Kindness Curriculum (KC):* The mindfulness-based SEL component of this curriculum is an adaptation of the KC developed by the Healthy Minds Innovations, Inc - Center for Investigating Healthy Minds, University of Wisconsin-Madison, USA [38]. The KC is a mindfulness-based SEL curriculum designed for preschool children (4 to 6 years) which aims at developing the *Awareness*, *Connection*, and *Insight* P-WB skills. Activities are detailed in a manual, are structured and progressive, and are based on books, music, and physical activities related to self-awareness, empathy, gratitude, and kindness [38].
  The KC has undergone prior scientific evaluation, demonstrating that it leads to positive outcomes on various indicators—sharing proneness, teacher-reported social competences, cognitive flexibility, self-regulation, and grades in preschool children—when delivered by trained mindfulness instructors [38]. Another pilot RCT tested delivery by teachers instead of instructors and found that preschool children that were allocated to an adapted version of the KC showed better attentional focus and self-regulatory skills compared to children in an as-usual condition, although no change in empathy or compassion were observed [61].
  French adaptation was undergone during a pilot study conducted with 8 kindergarten teachers between September 2019 and June 2021 [21]. Teachers were trained to implement the program and qualitative data (focus groups and personal interviews) were collected to assess program relevance and difficulties in implementation. Adaptation included modifying five books of the original program which were not available in France. Books targeting the same topics were found and tested. While the original version of the KC delivered by mindfulness instructors was designed to be implemented across 12 weeks with two 20–30-min sessions per week, our adapted version divided the KC lessons over the course of 2 years, from the beginning of pre-K to the end of kindergarten, with two 20–25-min sessions per week during a total of 48 weeks. Adaptations followed teacher requests during piloting, who agreed that sessions were excessively long, that the program would benefit from having the possibility to revise previous concepts and examine multiple times each topic. As a result, some lessons were split in two (e.g., the third lesson of theme 1 has been split in two parts, one dedicated to the “seeds of kindness” and the second part dedicated to the “follow me” game).
  The adapted French version retained the original division into eight global themes, with each theme divided into three lessons. The first themes (“theme 1: “mindful bodies and planting seeds of kindness”, theme 2: “I feel emotion of the inside”, theme 3: “how I feel on the inside shows on the outside”, and theme 4: “taking care of strong emotions on the inside and outside”) are covered during the Pre-K year, and the last themes (theme 5: “calming and working out problems”, theme 6: “gratitude”, theme 7: “all people depend on each other and the earth” and theme 8: “gratitude and caring for our world”) are covered during kindergarten after revising core Pre-K lessons. In the present evaluation, only the first 4 themes were covered (Pre-K year adaptation).
  As in the original program, each session is structured as follows: (1) *Introduction phase*. Teachers introduce the KC time, by initiating routine activities: reunion of children in circle, meditation bell ringing, and breathing exercises. (2) *Teaching phase*. Teacher introduces pupils with a new notion (e.g., pay attention, feelings, “peace wands” …), makes links between a previous lesson and the new one, reads a story, and asks questions about it (“why was the girl quiet?”). (3) *Active engagement phase*. Children put notions into practice and realize activities: role-playing (“peace wands”), planting seeds together, playing imitation game, practicing breathing exercises with bean bag animals …. (4) *Closing phase*. Teacher closes the lesson with a take-home message.
  Teachers are asked to deliver at least two 20–25-min lessons of the KC per week.
2. *Yoga-based ritualized activities:* A yoga-based program was developed in collaboration with a professional yoga instructor and a kindergarten teacher experienced in delivering yoga activities to her classrooms. Teachers received a manual along with 30 “yoga cards” to implement activities devoted to six categories: warm-up, auto-massaging, exercises while sitting down (including breathing exercises), exercises while standing (postures), relaxation. Exercises were tailored during the pilot-study to be accessible for teachers without prior yoga practice.
  Teachers were given freedom to implement different activities in different sessions, but a typical order for one session was proposed, with the following steps:
  *Warm-up phase*. Children stretch themselves.
  *Massage phase*. Children massage themselves different parts of their body. Progressively, teachers introduce more body-linked vocabulary (e.g., “arm,” “elbow,” “forearm,” “wrist”…).
  *Postures and controlled-breathing phase*. Children learn to reproduce yoga postures (seated and standing) adapted for children and modeled by teachers. They also learn to become aware of their breathing and to control it.
  *Relaxation phase*. Children focus themselves on breathing and/or inside their body.
  Exercise level increases progressively throughout the year, with the first sequences mostly focused on introducing the activity and familiarizing children with it, using easy exercises and vocabulary.
  Teachers are asked to deliver at least 20–25 min of yoga-based activities per week, in a minimum of one session, and are encouraged to deliver everyday sessions to ritualize practice.
3. *Emotion circle time:* A ritualized emotion circle time developed at Mons University, Belgium, completed the program [43]. Emotion circle time was added to the program to target the *Insight* and *Connection* components of P-WB. It follows a ritualized procedure to explore children’s emotions regarding a particular time of the day (typically recess) and finds solutions for children exposed to negative emotions. Children are asked to express their emotions individually by choosing between diverse “smiley” faces on a card (“happy,” “sad,” “fearful,” or “angry”). Then, teachers preferentially ask children who chose an emotion with negative valence to explain why they are feeling this. Three rules are used and recalled by children at the beginning of each session: (1) emotion cannot be denied by others; children use “I” statements and their feeling cannot be contradicted (e.g., “I feel… because…”); (2) teacher distribute speaking time (e.g., by giving and taking back a “talking stick”) and children speak one at a time; (3) children do not name or accuse others (they are asked to use the “someone” pronoun)—i.e., focus is put on finding solutions altogether and not on accusing others. Children discuss between themselves and with the teacher what could be implemented to help this child feel better. Finally, teachers remind children that this situation will be reexamined during the next session to monitor the evolution until the problem is resolved.
  Teachers are asked to deliver at least 20–25 min of emotion circle time per week, in a minimum of one session, and are encouraged to deliver everyday sessions to ritualize practice.

#### Criteria for discontinuing or modifying allocated interventions {11b}

The intervention is discontinued for children only when children change schools or classrooms during the year. The protocol does not allow modifying the allocated intervention.

#### Strategies to improve adherence to interventions {11c}

No specific strategy is implemented to improve adherence. However, to control and improve fidelity to intervention, implementation notebooks are distributed to teachers in the intervention group at the beginning of the program. Each week, teachers are asked to write down the curriculum activities that have been implemented each day, the duration of activities and to add comments if necessary. In the middle of the school year, teachers are also asked to record themselves with a sound recorder while implementing curriculum activities: in total, two sessions per activity (kindness curriculum, yoga-based activities, and emotion circle time) are registered and transferred to the principal investigators [TV, OC] to evaluate implementation fidelity. Fidelity is assessed for each record by two independent evaluators following a 3-question list inspired by Humphrey et al. [44]: (1) *Objectives: To what extent does the teacher cover the general and specific objectives of the lesson?* (2) *Structure: To what extent does the teacher follow the structure and sequence of activities outlined in the instruction manual?*; (3) *Content: How closely does the teacher adhere to the guidance manual when teaching the core activities of the lesson?*. Evaluators rate each question on a qualitative scale from 1 (= “Insufficient”) to 5 (= “Very satisfactory”). For each activity, scores for each question in each record for each evaluator are averaged into a total fidelity score on a 1-to-5 scale.

#### Relevant concomitant care permitted or prohibited during the trial {11d}

Treatment of all sorts for children is not controlled in this trial. It is therefore possible for children to begin or stop a drug treatment or a psychological intervention during the trial. At the end of the first and second year of trial, all teachers (intervention group and control group) are asked if they participated in other trainings that aim to develop P-WB or SEC in children over the course of the year.

#### Provisions for post-trial care {30}

N/A. No provision for ancillary or post-trial care is provided.

### Outcomes {12}

Our primary objective is to assess curriculum effects on P-WB-Skills measures of *Connection* and *Insight*, on P-WB measures of *Engagement* and *Positive Relationships*, and on *Self-management*. Our secondary objective is to document curriculum impact on measures of mental health, executive functioning, and school performance. All outcomes are evaluated at baseline (pre-intervention in Pre-K), at the end of the first year of intervention (Pre-K), and at follow-up (kindergarten), except for school performance which is only evaluated at follow-up (1st grade).

*1.a. Primary outcomes: P-WB skills, P-WB, and self-management measures*

**Table:** 

***Component***	***Type of measure***	***Name of tool***	***Variable***
*Connection*	Questionnaire	PKBS-social skills	Social interaction subscale
			Cooperation with peers subscale
	Task	Sharing task	Sharing proneness score
	Task	Peer acceptance task	Peer acceptance score
*Insight*	Task	Challenging situation task	Adaptive response score
	Task	Emotional matching task	Expressive emotional knowledge score
*Engagement*	Standardized observation	InClass System	Positive engagement with teacher
			Positive engagement with tasks
*Positive relationships*	Questionnaire	PKBS-social skills	Agreeableness with peers subscale
	Questionnaire	STRS-short form	Closeness score
	Standardized observation	InClass System	Positive engagement with peers
*Self-management*	Questionnaire	PKBS-social skills	Autonomy subscale
			Compliance subscale
	Questionnaire	STRS-short form	Conflict score
	Standardized observation	InClass System	Negative classroom engagement score

*Abbreviations: InClass*, Individualized Classroom Assessment Scoring System; *PKBS*, Preschool and Kindergarten Behavior Scale; *STRS*, student-teacher relationship scale-short form

*1.b. Secondary outcomes: mental health, executive functioning, and school performance*

**Table:** 

***Component***	***Type of measure***	***Name of tool***	***Variable***
*Mental health*	Questionnaire	SDQ	Total difficulties score
	Questionnaire	SDQ	Impact score
*Executive functioning*	Task	EF battery, House, Pick the picture & Something the Same	Working Memory Span Score 1 (House) Working Memory Span Score 2 (Pick the picture) Cognitive Flexibility Score
*School performance*	National testing evaluations in math and reading	évaluation nationale *EVALAIDE*	

*Abbreviations: EF*, executive functioning; *SDQ*, Strengths and Difficulties Questionnaire

### Participant timeline {13}

Outcome measures are collected before the start of the intervention (pre-K; October-November 2021, T0), at the end of the first year (May-June 2022; pre-K; T1), at the beginning of the second year for kindergarten teachers’ characteristics only (October 2022; kindergarten; T2), at the end of the second year (May-June 2023; kindergarten; T3), and at follow-up for school performance only (1st grade; September 2023 and March 2024; T4). Data are collected directly by principal investigators [OC] from computerized forms (questionnaires and scales), and by trained evaluators recruited for the study for tasks and observations. Tasks and observations take place in schools—in the classroom, hallways, and recess for observations, and in a dedicated room for experimental tasks.

#### Enrolment

Children’s eligibility was determined in September 2021. Information and consent refusal forms were given to parents. Lists of final eligible children were established for each class.

#### Visits and data collection

##### Before intervention (T0)

Questionnaires assessing baseline children characteristics are sent to teachers and returned to principal investigators by e-mail. Evaluators visit each school to observe children and administer tasks. Visits take approximately 3 school days for a maximum of 12 children examined in each classroom. Questionnaires assessing teacher characteristics are sent to all teachers by e-mail at the beginning of the year.

##### End of first year (T1)

At the end of the first year (Pre-K), questionnaires assessing T1 children’s characteristics are sent again to teacher and evaluators re-evaluate children, following the same procedure.

##### End of second year (T3)

All outcomes assessed at T0 and T1 are reassessed.

##### Follow-up (T4)

In France, the Ministry of Education organizes each year since 2017 an evaluation of the mathematics and reading skills of all Grade 1 and Grade 2 students. We will use these tests to assess the long-term effects of the curriculum in mathematics and reading in Grade 1 and Grade 2 (Table 1).

**Table 1:**
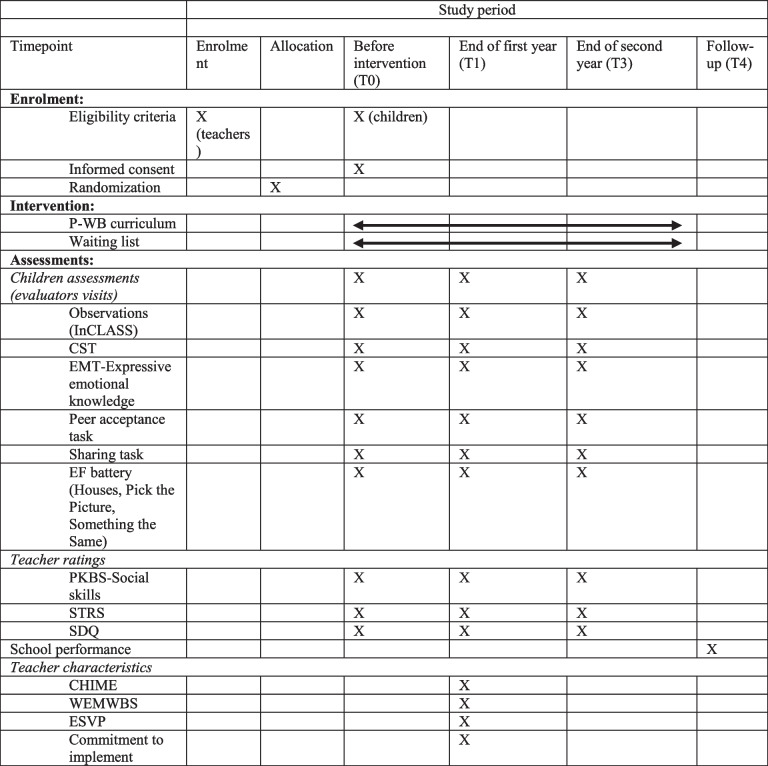
Data acquisition and trial timeline

*Abbreviations*: *CHIME* Comprehensive Inventory of Mindfulness Experience, *CST* Challenging Situations Task, *EF battery* executive functioning battery, *EMT* emotion matching task, *ESVP* Satisfaction with professional life scale, *InCLASS* Individualized Classroom Assessment Scoring System, *PKBS* Preschool and Kindergarten Behavior Scale, *STRS* Student-Teacher Relationship Scale, *SDQ* Strengths and Difficulties Questionnaire, *WEMWBS* Warwick-Edinburgh Mental Well-Being Scale

### Sample size {14}

A previous meta-analysis of SEL interventions in preschool children found effects in the medium range (mean of 0.35 standard deviations) for socio-emotional outcomes [56] while intra-cluster coefficient (hereafter ICC) for socio-emotional measures are typically between 0.02 and 0.2 for teacher-reported outcomes [23, 32, 38] and inferior to 0.1 for behavioral tasks such as executive functioning tasks [38, 40].

We performed power calculations in order to estimate the number of classrooms required to observe a minimum detectable effect size (MDES) of 0.35 standard deviations, as expected from the meta-analyses of Murano et al. [56]. We followed Bloom [12] to perform rigorous clustered-design power calculations and took into account the various parameters that can affect the sample size required to detect a specific MDES. In particular, we took into account various levels of the ICC, attrition rate, and augmented power coming from control variables. Overall, for a total number of 10 observed students per classroom, we needed to recruit at least 55 classrooms to observe an MDES of 0.35 standard deviations.

### Recruitment {15}

Teachers (pre-K level) were recruited between April and June 2021. Information about the study was distributed to public school principals and teachers through the departmental direction of education services (*Direction des services départementaux de l'Éducation nationale* (DSDEN)) using emails. Oral presentations of the study were then organized within each municipality. Interested teachers contacted the principal investigator (TV) of the study through emails and were accepted until sample completion.

Children were recruited in each classroom by the teachers, who transmitted information to parents regarding the study and collected consent to participate. As we targeted a final number of 10 observed students per classroom, a sample of 12 students was targeted in each classroom for the experimental and observational part of the protocol, to take into account the fact that recruitment in classrooms with a mixture of children from Pre-K and kindergarten levels may be reduced. Teachers provided one of the investigators in charge [OC] with a list of students fitting the inclusion and exclusion criteria. The principal investigator then randomly selected 12 children from that list and added two children who were put on a waiting-list, in case one of the 12 children initially targeted would be missing on the experimental visit day. Questionnaire data was collected for all children including children on the waiting-list. In order to also collect data on children with severe behavior problems who had to be excluded from the experimental and observation protocol (*specific exclusion criteria*), questionnaire data on two more children with severe behavior problems fulfilling the other inclusion criteria were collected in each classroom whenever possible.

## Assignment of interventions: allocation

### Sequence generation {16a}

Randomization was performed after having recruited 64 classes. Allocation sequence was generated online on the website randomizer.org by one of the principal investigators [TV] who will not be in charge of data collection, assessment, and analysis. Investigators in charge [OC, QD] are blinded to group allocation.

### Concealment mechanism {16b}

Allocation sequence was generated by a principal investigator [TV] who is not in charge of data analysis.

### Implementation {16c}

One of the principal investigators [TV] generated the allocation sequence, enrolled teachers, and assigned teachers to interventions.

## Assignment of interventions: blinding

### Who will be blinded {17a}

Investigators in charge of data analysis [OC, QD] are blinded regarding group allocations. Due to the nature of the experiment, teachers cannot be blinded regarding group status, as they deliver the program themselves. Evaluators assessing children in classrooms based on the *InClass* observation system and on experimental tasks are blinded both regarding group allocations and regarding the nature of the ongoing study. Blindness of evaluators regarding allocations and study nature is assessed at the end of the school year. Teachers in the classrooms are asked both orally and by written instructions not to communicate with evaluators regarding the study or the program, and had to answer to an email stating that they had received and understood these instructions.

### Procedure for unblinding if needed {17b}

N/A. No procedure was prepared for group unblinding.

## Data collection and management

### Plans for assessment and collection of outcomes {18a}

Computerized teacher rating questionnaires (PKBS-Social Skills, STRS, SDQ) and questionnaires evaluating teacher characteristics (CHIME, WEMWBS, ESVP, Commitment measure) are collected in electronic form by a principal investigator blind to group allocation [OC]. Data encoding is verified by OC to detect software errors when encoding form data into a spreadsheet.

Tasks and observations are administered by trained evaluators, students in their third or fourth year of psychological or education sciences studies, who receive an intensive 3-day training and pass a certification test prior to school visits. During the first day of training, evaluators work by pairs through role-playing and learn how to administer each task, based on a manual with explicit instructions. They are also given advice for task processing and handling children. Ability to carry on reliably two randomly chosen tasks is evaluated by the principal investigators 3 days later. On the second and third day, evaluators are trained by certified trainers [OC, TV, VK] to use the InCLASS observation tool, using the standardized InCLASS training course. During this course, each InCLASS dimension is described to trainees with video examples, and they watch, code, and discuss five training video clips. To validate InCLASS training, evaluators are required to code five reliability clips independently and to obtain a correct response proportion of a least 80% for all InCLASS dimensions (and at least 3 correct responses for each dimension). Evaluators who are not reliable after this first test are given a second chance as they are asked to code five extra reliability clips. Evaluators who are still not reliable after this second step are excluded. Inter-rater reliability during the study is measured by collecting double-coding for one child during each visit, resulting in 12.5% double-coded InCLASS observations.

When evaluating children, evaluators are referring themselves to detailed instruction manuals (InCLASS + experimental tasks). InCLASS manual describes each dimension in detail, and provides detailed examples of coding for each dimension. Experimental task manual describes material and process of each task, with detailed verbatims. Tasks are conducted in the following order: EF battery (two randomly assigned tasks from three possible tasks: Something the Same, Houses, Pick the Picture), Peer acceptance task, Sharing task, CST task, EMT task.

Data collections are conducted during approx. 3 school days (for 12 children) by two evaluators in each school. Mornings are dedicated to inCLASS observations. Evaluators conduct four (2 observations x 2 evaluators) observation cycles (10-min observation + 5-min coding), resulting in 4 observations per child. Observation sessions last approx. 3 h. Tasks sessions are conducted in the afternoons and last approx. 40 min per child.

Evaluations take place before intervention (T0; October–November 2021), at the end of the first year of trial (T1, May–June 2022), and at the end of the second year of trial (T3, May–June 2023). Data collection forms are available by request to the principal investigators [TV,OC].

Description of the tools used and variables retained:

1. School performance
  Indicators of school performance are obtained 2 years after the beginning of the intervention, based on national evaluations taking place in the first and second years of primary school in France (*EVALAIDE* in Grade 1 and Grade 2). These evaluations target various literacy and math skills: reading out loud words, reading out loud text, understanding phrases while reading them, writing syllables, writing words, operating phonemes, recognizing letters, comparing letters, knowing letters’ names and sounds, understanding words, understanding phrases, understanding texts, reading numbers, writing numbers, visualizing numbers, comparing numbers, ordering numbers in sequence, resolving math problems, calculating mentally, adding numbers, subtracting numbers, and reproducing geometrical forms. *Mathematic skills, Reading skills*: based on these tests, we will create two outcomes corresponding to mathematics and reading skills.
2. Standardized observations
  *Individualized Classroom Assessment Scoring System (InCLASS):* The InCLASS is a standardized naturalistic observation tool analyzing 3 to 5 years old children’s interactions in a classroom context [34]. It comprises 10 dimensions: (1) positive engagement with the teacher (which comprises the following indicators: attunement to the teacher, proximity seeking, and shared positive affect with teacher), (2) communication with the teacher (conversation initiated and maintained with the teacher, and variety of speech with the teacher), (3) teacher conflict (aggression, negative affect, attention-seeking behaviors, and non-compliance toward the teacher), (4) peer sociability (proximity seeking, shared positive affect, cooperation toward peers, and popularity), (5) peer communication (conversation initiated and maintained with peers, and variety of speech with peers), (6) peer assertiveness (positive initiation of interactions with peers and leadership toward peers), (7) peer conflict (aggression, negative affect, attention-seeking and confrontation toward peers), (8) engagement with tasks (sustained attention and active engagement in classroom activities), (9) self-reliance (personal initiative and independence in classroom activities), and (10) behavior control (patience, activity level matching expectations, and physical awareness) [13]. These dimensions have been grouped into four domains in a previous study using confirmatory factor analyses [13]: positive engagement with teachers (grouping positive engagement and communication with teacher), positive engagement with peers (peer sociability, peer communication, and peer assertiveness), positive engagement with tasks (engagement with tasks and self-reliance), and negative classroom engagement (teacher conflict, peer conflict, and the reversed score of behavior control). This tool has demonstrated solid inter-rater reliability, construct validity, and criterion validity [34]. *Positive engagement with teachers score, Positive engagement with peers score, Positive engagement with tasks score, Negative classroom engagement score:* Each dimension is rated on a 7-point scale, with higher score indicating higher frequency and/or quality of behavior within a dimension. Data from each of the observation cycles—collected by each data collector—are averaged to obtain final dimension scores. Mean score for the four domains are then calculated by averaging final dimensions scores within each of the four domains. Inter-rater agreement is calculated based on 12.5% of all observations, where the two data collectors independently observe the same child.
3. Tasks
  *Challenging Situations task (CST):* An app-version of the full CST task is used to assess children’s emotional response in challenging interpersonal problems and ability to solve interpersonal problems [28]). Situation, emotion vignettes, and behavioral response vignettes are displayed on a tablet and responses are automatically registered. Emotional responses are composed of four different choices: happy, indifferent (“just ok”), sad, and angry. Behavioral responses are composed of four types of choices: a prosocial choice, an aggressive choice, an avoidant choice, and a dysregulated (“crying”) choice [27]. Examiners describe each situation picture and ask what the child would do in such a situation, by presenting response picture choices and asking the child to point at the picture corresponding to their emotion/behavior when/if the situation happens. Previous studies suggested that choosing sad emotion and prosocial behaviors on the CST constitutes adaptive behaviors that are linked with emotion knowledge and peer acceptance [28], and predicts classroom adjustment, pre-academic literacy skills, kindergarten readiness and academic achievement [26, 27, 81]. On the opposite, choosing aggressive response is associated with poor peer acceptance [28], poor classroom adjustment, and poor academic readiness [27]. *Adaptive responses score, Aggressive behavior score:* Along with previous studies [26, 81], the proportion of adaptive responses (Sad emotion + Prosocial behavior” responses) and aggressive behavior responses across the six vignettes (ranging from 0 to 6) are calculated.
  *Emotion matching task-expressive knowledge:* The expressive knowledge sub-task of the Emotional Matching Task (EMT) measures 3 to 6- years-old children expressive emotion knowledge – i.e., the ability to recognize and label expressions of others’ emotions (based on Izard test [46, 55];). A sample of 12 colored photographs representing ethnically-diverse children with emotional facial expression are presented on a tablet screen: happiness, sadness, fear/surprise, anger, and mixed (anger/sadness). Children are asked each time what the child on the picture is feeling [55]. The EMT has demonstrated good criterion validity, strong reliability and construct validity [55], and preliminary evidence for cross-cultural validity has been found [3]. *Expressive emotional knowledge score:* Each child verbal responses are assigned to a score of 0 (= incorrect response), 1 (=accepted response), or 2 (=correct response). Accepted verbal responses are pre-determined and listed in a table before test sessions using the instruction manual. All items are summed to obtain the total score, with higher score indicating better expressive emotion knowledge.
  *Executive functioning battery:* We use an app-version of the EF battery (“*EF Touch*”), a battery of six EF tasks designed for preschool children from 3 to 5 years, which was found to show good criterion validity [83]. Each response is automatically registered on a computer communicating with the tablet. Analyses of dimensionality show that performance on EF tasks is best characterized by a single EF factor [83]. The Houses, Pick the Picture and Something the Same tasks, when combined, best approximate an EF latent variable underlying performance on all the six tasks [84]. We therefore selected these three tasks to assess children’s EF within a single EF latent factor model. Finally, following the suggestion of Willoughby et al. [84], only two randomly selected tasks out of three are administered to each child using a planned missing design, in order not to overload attention capacities of children and to reduce global test burden. Score for the missing parameter is estimated through an imputation procedure.
  *Houses game (also named Working Memory Span game):* This task assesses working memory span. Children are presented with houses in which are located a line-drawing animal and a color dot. They are asked to name both the animals and the colors in each of the houses. Then, animals and color dots disappear from houses. Children have to recall either which animal *or* which color was in the target house, thus holding in mind two pieces of information and activating one of them (i.e., animal name) while overcoming interferences from the other (i.e., color name) [84]. Task becomes increasingly difficult as the number of houses on the screen increases (from one to three houses).
  *Pick the Picture (PTP) game:* This is a self-ordered pointing task assessing working memory. In this task, children are asked to touch once each picture appearing on the screen, so that all of the pictures “get a turn” [84]. Between each touch, location of pictures is changed in a randomized order. Difficulty of the task increases as the number of pictures increases (from two to six pictures).
  *Something’s the Same (STS) game:* This task evaluates cognitive flexibility. For each item, children are asked to shift their attention from one dimension of similarity to another dimension of similarity [84]. Initially, they are presented with two pictures that share one dimension of similarity (e.g., color, shape, size, etc.). Then, a third picture is presented, and children have to tell how this new picture is similar to one of the original pictures. This last picture always shares a different dimension of similarity with one of the original pictures. In the second part of the game, all pictures are presented at once and children have to identify two different dimensions of similarity.
  *Working memory span score 1 (Houses), Working memory span score 2 (Pick the picture), Cognitive Flexibility Score*: for each EF battery game, each response is coded into a dichotomous variable (0 = incorrect response, 1 = correct response). The total score is a proportion ranging from 0 to 1, with 0 indicating no items correct and 1 indicating all items are correct.
  *Peer acceptance task:* The peer acceptance task measures how a particular child is accepted and liked by his peers in his/her class. It is a peer rating measure inspired by Asher et al. (1979) sociometric procedure [14]. Children are presented with photos of their classmates. After identifying classmates on these photos, they are asked to sort each photo of classmates into three envelopes: happy smiley for “I really like to play with this child”; neutral smiley for “I kind of like to play with this child”; unhappy smiley for “I don’t like to play with this child”. Answers are coded 3, 2, and 1 respectively. This procedure shows adequate reliability and validity [14]. *Peer acceptance score*: After testing each child, we assess how many times a child photo is put in each envelope: a mean score is calculated by summing up the score on each trial and dividing by the number of children in the classroom minus one. The final score indicates how much a child is accepted by his peers, with higher scores indicating higher levels of acceptance.
  *Sharing task:* This task evaluates child sharing. The sharing task used in this study is a sub-section of the task designed by Flook et al. [38], divided into two trials. For each trial, children are presented with 10 stickers. They are told that they can keep as many stickers as they want for themselves and share as many as they want with another child. They then separate stickers between two different envelopes (identified with the photo of the assessed child and the photo of the other child). In the first trial, the other child is the one identified by the assessed child as his/her most-liked classmate, and in the second trial, the other child is the one identified as the least-liked classmate. Sharing ability has been shown to differ between groups that received an SEL intervention compared to control group [38]. *Sharing proneness score*: 20 minus the total number of stickers (ranging from 0 to 20) put in the “me” envelope across the two trials is calculated. Higher scores indicate higher levels of sharing tendencies.
4. Teacher-rated questionnaires
  *Commitment to implement:* Teachers in the intervention group are asked about their commitment to implement the program using a single item: “I feel motivated to use the program/strategies in my classroom”. Previous study indicates that this item is a valid indicator of teacher commitment to implement various evidence-based programs, regarding construct validity and convergent validity with other measures of commitment and with commitment predictors [22]. Teachers in the intervention group are also asked whether they liked the training, whether they found it useful, whether they found the content of the program useful, whether they liked the content of the program, and whether their students liked the content of the program. These items are rated on a 5-point Likert scale (from 1= “strongly disagree” to 5 = “strongly agree”). *Commitment score*: We will construct a standardized commitment score based on these previous items. Higher score indicates higher commitment to implement. As the psychometric properties of this score have not been validated, we will check the internal validity of the constructed score using Cronbach’s Alpha.
  *Preschool and kindergarten behavior scale (PKBS)- Social skills:* The PKBS social skills [54] is a teacher-rated scale assessing preschool and kindergarten children’s social skills. The French version of the scale is divided into 5 subscales [20]: The first subscale, “social interaction’ consists of 10 items that reflect behaviors and attitudes necessary to develop and maintain good relationships and friendship with others (such as defend others’ rights, helping others), “participates in classroom or family discussions,” “shows affection for other children,” or “tries to understand another child’s behavior.” The second subscale consists of six items and reflects components of “agreeableness with peers,” such as “plays with several different children,” “makes friend easily,” or “smiles and laughs with other children.” The third subscale, “compliance,” relates to respect for adult authority and social norms, such as “follows instructions from adults,” “follows rules,” or “uses free time in an acceptable way.” The fourth subscale, “cooperation with peers,” describes behaviors such as “shares toys and other belongings” or “gives in or compromises with peers when appropriate.” Finally, the fifth factor, labeled “autonomy,” is composed of items such as “works or plays independently,” “adapts well to different environments,” or “attempts new tasks before asking for help.” *Total PKBS Social Skills Score, PKBS Social interaction score, PKBS Agreeableness with peers score, PKBS Compliance score, PKBS Cooperation with peers scores, PKBS Autonomy score; PKBS functional level categorical score*: The 34 items are rated on a 4-point Likert scale ranging from 0 (= Never) to 3 (= Often). Total raw scores and raw scores for each subscale are calculated by adding scores of each item, with higher scores indicating better social skills. For 5–6-year-old children, total raw scores are divided into 4 functional level categories based on child age: “high functioning” (scores 95 to 102), “average” (scores 76 to 94), “moderate deficit” (scores 59 to 75), and “significant deficit” (scores 0 to 58) to compute a functional level categorical variable.
  *Strengths and difficulties questionnaire (SDQ)-extended teacher version*: The SDQ-extended teacher version is a scale used to measure children’s externalized symptoms, internalized symptoms, and pro-social behaviors [42]. It is divided into five factors of five items each: emotional symptoms, conduct problems, hyperactivity-inattention, peer problems, and prosocial behavior. At the end of the questionnaire, 4 items explore impacts of difficulties (overall distress, social impairment, learning impairment, and burden for teacher and classroom) [41]. The SDQ displays satisfactory reliability and validity [42] and although its factor structure in French language remains uncertain [15, 16], the SDQ French version shows satisfactory reliability and content validity for the total difficulties score [16]. *SDQ total difficulties score, SDQ impact score*: The 25 items are statements rated on a 3-point Likert scale indicating to what extent each statement applies to a target child, with 0 = Not true, 1 = Somewhat true, 2 = Certainly true. All factors except Prosocial behavior are summed in a Total difficulties score. Items relative to impact of difficulties are coded from 0 to 2, with 0 corresponding to no impact or little impact, 1 corresponding to “Somewhat impactful”, and 2 to “Very impactful”. An impact total score ranging from 0 to 8 is then generated by summing these items. Higher scores indicate higher levels of difficulties and higher level of impact.
  *Student-teacher relationship scale-short form (STRS):* The STRS-short form is a teacher-rated scale exploring teachers’ perspective on their relationship with a designed child in their classroom [60]. The scale comprises two distinct factors, one measuring the degree to which teacher-child relationship is characterized by warmth, positive emotions and open communication (closeness), and the other measuring the degree to which the relationship is characterized by negative emotions and interactions (conflict). This scale possesses excellent psychometric properties across multiple samples [60]. The validation of the French version is ongoing [21]. *STRS Closeness score, STRS Conflict score:* The 15 items (8 items assessing Closeness, 7 items assessing Conflict) are scored using a 5-point Likert scale ranging from 1 (= Definitely does not apply) to 5 (= definitely applies). Total scores are then calculated for each factor.
  Teacher personal well-being P-WB:
  *Comprehensive inventory of mindfulness experience (CHIME):* The Comprehensive Inventory of Mindfulness Experience (CHIME) is a self-report multidimensional scale measuring “dispositional” (or “trait”) mindfulness in adults with or without knowledge or previous contact with mindfulness [9, 53]. It captures the following eight dimensions of “trait” mindfulness: awareness of internal experience, awareness of external experience, acting aware, self-acceptance and non-judgment, non-reactivity, openness and non-avoidance, ability to relativize, and insight. The CHIME exhibited good reliability and satisfactory construct validity [9]. The validated French version of the scale used in this study showed highly satisfactory psychometric properties as well [71]. *CHIME Total score:* The 37 items are rated on a 6-point Likert scale (from 1 = “fully applies” to 6 “does not apply at all”). Total score is calculated by inverting each item score and adding all inverted score. Higher scores indicate higher proneness to experience mindfulness in everyday life.
  *Warwick-Edinburgh mental well-being scale (WEMWBS):* The Warwick-Edinburgh mental well-being scale (WEMWBS) is self-report single-factor measure composed of positively worded items related to positive mental health and well-being [74]. It covers a wide-range of concepts associated with positive mental health, including hedonic and eudemonic well-being, satisfying personal relationship, and positive functioning. The original version displayed good content validity and reliability [74], and the French version used in this study showed good internal consistency, stability and construct validity [77]. *WEMWBS Total score*: The 14 items are rated on a 5-point Likert scale (from 1= “never” to 5 = “always”), with high score indicating higher mental well-being.
  *Satisfaction with professional life scale (Echelle de Satisfaction de Vie Professionnelle; ESVP):* The ESVP scale is a unidimensional French short self-report measure of general professional life satisfaction derived from the Satisfaction with Life scale from Diener et al. (1985) [29, 39]. It displays adequate level of internal consistency and temporal stability, and good construct validity [39]. *ESVP total score*: The 5 items are rated on a 7-point Likert scale (from 1= “strongly disagree” to 7 = “strongly agree”), with higher scores indicating higher professional life satisfaction.

### Plans to promote participant retention and complete follow-up {18b}

No specific plan was designed to promote participant retention and complete follow-up.

### Data management {19}

The study complies with the European General Data Protection Regulation (GDPR), and data management is under control of Paris-Lumières University local GDPR referent. All parents receive an information note regarding who will have access to data, who is responsible for data management, and regarding the fact that data will be managed in accordance with the GDPR French law. They are also informed that the data provided will be treated confidentially and that in published reports the results will be reported anonymously and at a group level, meaning that it will not be possible to identify any individual or attribute any information to them. Data quality for data collected on paper will be checked by randomly double-checking 20% of data entry. In case of data error, all data will be double-checked.

### Confidentiality {27}

Identity of children participating in the study has been collected from the teachers of the classes involved on paper list by the principal investigators [TV, OC]. All participants have received an anonymous identity code and correspondence between personal identity and code is stored on a file only accessible to the principal investigators [TV, OC].

### Plans for collection, laboratory evaluation, and storage of biological specimens for genetic or molecular analysis in this trial/future use {33}

N/A. This trial will not involve collection and storage of biological specimens for genetic or molecular analysis.

## Statistical methods

### Statistical methods for primary and secondary outcomes {20a}

We will evaluate the effects of the intervention on teachers’ P-WB and on children’s P-WB, self-management, mental health, executive functioning, and school performance. To estimate the effects on all the outcomes listed in the “Outcomes {12}” section, we will run an OLS regression of that outcome on a dummy variable indicating whether the teacher was assigned to the intervention. When necessary, we will add to the model a vector of pre-determined covariates that are unaffected by the treatment such as socio-demographic characteristics or the level of the outcome measured before the start of the intervention. For each estimation, we will cluster the standard errors at the unit of the randomization. In addition to the intention-to-treat (ITT) estimates, we will also compute the treatment-on-the-treated (ToT) estimates using the so-called Wald estimator based on the actual take-up of the intervention according to the measures of fidelity of implementation.

In order to take into account multiple testing issues, we will follow the methodology given in Anderson [5]. First, we will group outcomes into families, and, in each family, we will construct the so-called standardized treatment effect with weights accounting for the variances and covariances of the outcomes, in order to maximize the information captured by the weighted average. Second for each estimation within each family, we will report both the unadjusted *p*-value of the coefficient of the treatment variable, and the *p-*value adjusted for control of the false discovery rate [8].

### Interim analyses {21b}

N/A. No interim analyses are planned.

### Methods for additional analyses (e.g., subgroup analyses) {20b}

We will estimate the effects of the intervention on the children’s and teachers’ outcomes listed in the “Outcomes {12}” section based on the following characteristics:

- Girls (for the children)
- Level of experience of the teachers (according to the median)
- Age of the teachers (according to the median)
- Initial levels of EF, connection, and problem behaviors of the children
- Multi-level classes
- Teachers predicted to have a high degree of commitment with the intervention by a machine learning model

According to the data collection plan detailed in this paper, we will ask questions about treatment-group teachers’ commitment with the intervention (see Plans for assessment and collection of outcomes {18a} section) and will average teachers’ answers into a standardized score of their commitment with the intervention. Then, we will run a Lasso regression [7] of that score on all the socio-demographic variables and the outcomes listed in the “Outcomes {12}” section measured at baseline, on the square of those variables, and on the products of all the pairs of variables. We will then use the Lasso regression to predict the commitment score of every teacher included in the experiment, hence including teachers in the control group. Finally, teachers predicted to have a high degree of commitment with the intervention will be those with a predicted score above the median. To compute predicted commitment with the intervention for treatment group teachers, we will use a leave-one-out method, as suggested by Abadie et al. [1]. It may however be the case that we are not able to predict the teachers’ commitment score very well, in which case, undertaking that third subgroup analysis would not be informative. If the Lasso regression does not select any variable, or if the R2 of the OLS regression of the commitment score on all the variables selected by the Lasso regression is below 0.1, we will not conduct that subgroup analysis.

### Methods in analysis to handle protocol non-adherence and any statistical methods to handle missing data {20c}

We will measure non-adherence to the random assignment and to the implementation of the curriculum thanks to the different data collected over the course of the school year. As we estimate intention-to-treat effects, we respect the initial random assignment of the teachers to the treatment or the control groups, whatever of the adherence status to the initial assignment or the implementation fidelity.

In the Lasso regressions and in the final regressions where the effects of the treatment is estimated, missing values of the control variables will be replaced by the mean of these controls, and for each control, an indicator for observations for which the control is missing will be included in the regression. We will not impute missing values for outcome variables.

### Plans to give access to the full protocol, participant-level data, and statistical code {31c}

We will publish the full protocol, anonymous data, and related statistical codes used to analyze the data and estimate the effects of the curriculum on the different outcomes.

## Oversight and monitoring

### Composition of the coordinating center and trial steering committee {5d}

Data monitoring is carried out by principal investigators: they ensure progress of research protocol, randomization, data collection, and visit organization. A research assistant helps principal investigators in corresponding with teachers and evaluators and conducts administrative tasks relative to trial (e.g., teacher and evaluator remuneration, material purchase, and preparation). Data analysis and statistics are carried out by one of the principal investigators [OC], with the help of an expert statistician from the J-PAL ([QD], Abdul Latif Jameel Poverty Action Lab). Another research assistant helps principal investigators with evaluators’ training course. Evaluators are supervised by a principal investigator during the 3-day training and evaluation and are regularly in contact by phone and emails with principal investigator during visit periods. Principal investigators meet regularly to ensure and control trial progress and are in constant contact by emails.

### Composition of the data monitoring committee, its role, and reporting structure {21a}

An independent data monitoring committee has not been formed and no independent auditing will take place.

### Adverse event reporting and harms {22}

Due to the intervention nature, serious adverse events are not expected. Any adverse event that occurred will be reported in the manuscript describing trial results if they are directly related to intervention. Those adverse events will be monitored by principal investigators and addressed until resolution.

### Frequency and plans for auditing trial conduct {23}

Investigators accepts to comply with the regulatory requirements of the competent authority for a research audit. Audit may be carried out at any stage of the trial, from the protocol development to the publication of results and archival of data.

### Plans for communicating important protocol amendments to relevant parties (e.g., trial participants, ethical committees) {25}

No important protocol amendments are anticipated. All protocol modifications must be submitted to our Paris-Lumières ethics committee of the UFR SPSE, Psychological and Educational Sciences prior to implementation, and all participants must be informed of protocol changes.

## Dissemination plans {31a}

Regardless of magnitude or direction of effects, all relevant trial results will be submitted to scientific review for publication. No publication restriction is planned. Teachers will be informed through an online conference of the results of the trial, and parents can be informed of overall trial results by principal investigators [OC, TV] after a request by email.

## Discussion

This cluster randomized control trial examines the effect of a teacher-delivered mindfulness- and yoga-based socio-emotional learning curriculum in preschool children in France. Intervention is compared to a waiting-list control group. To our knowledge, this is the first study that evaluates rigorously the effect of a progressive P-WB intervention at school in France. One important operational issue encountered during the trial is the COVID-19 pandemic situation: during the school year, teachers may be personally ill and have to shut down their classes during several weeks; children may also not be able to attend school during some weeks if they are ill themselves. To monitor these possibilities, teachers will each be personally contacted by one principal investigator [TV] to monitor program implementation, and implementation notebooks will be reviewed at the end of the year with teachers to assess the influence of the sanitary situation on intervention delivery.

## Trial status

Protocol version: November 2021, third version.

Date of teacher recruitment: from June 2021 to September 2021

Date of children recruitment: from September 2021 to October 2021.

Due to organizational preferences for recruiting teachers and evaluators, implementing the program, and organizing the first session visits (from September to November 2021), the article is submitted after the end of recruitment. Importantly, the trial is still currently ongoing, as the first data have not entirely been computed yet, and the first results will not be analyzable before July 2022, at the end of the first year of intervention. Importantly, no analysis will be started as long as the presentation of the protocol has not been published.

## Supplementary Information

**Additional file 1.** Ethical Approval Document.

## Data Availability

Data will only be available to principal investigators.

## Abbreviations

CASEL: Collaborative for Academic, Social And Emotional Learning
CHIME: Comprehensive Inventory of Mindfulness Experience
CST: Challenging Situations Task
EF: Executive functioning
EMT: Emotion Matching Task
ESVP: Satisfaction with professional life scale
InCLASS: Individualized Classroom Assessment Scoring System
MBIs: Mindfulness-based interventions
PERMA: Positive emotion, Engagement, Relationship, Meaning and Accomplishment
PKBS: Preschool and Kindergarten Behavior Scale
P-WB: Psychological well-being
SEC: Socio-emotional competencies
STRS: Student-Teacher Relationship Scale
SDQ: Strengths and Difficulties Questionnaire
WEMWBS: Warwick-Edinburgh Mental Well-Being Scale
YBIs: Yoga-based interventions
