# Supplementary material for: Promoting psychological well-being in preschool children: study protocol for a randomized controlled trial of a mindfulness- and yoga-based socio-emotional learning intervention
Source: Trials. 2022 Dec 27;23:1050. doi: 10.1186/s13063-022-06979-2 (PMC9793351; doi:10.1186/s13063-022-06979-2)
Supplement: Supplementary file 1 — Additional file 1. Ethical Approval Document. [file 13063_2022_6979_MOESM1_ESM.pdf]

Université Paris Nanterre

UFR SPSE

Comité d'éthique de l'UFR SPSE
(Sciences Psychologiques et Sciences de l'Education)
Ethics committee of the UFR SPSE
(Psychological and Educational Sciences)

200, avenue de la république
92001 Cedex Nanterre
Contact : cer_spse@liste.parisnanterre.fr
N° d'avis : 04-n°1

Nanterre, le 8 avril 2020

**Les critères utilisés pour l'évaluation sont formalisées en français dans le « guide d'évaluation éthique d'un projet de recherche » disponible sur demande auprès du comité d'éthique de l'UFR SPSE.*

Le comité d'éthique de l'UFR SPSE de l'Université Paris Nanterre, après étude du projet de recherche, présenté par Thomas Villemonteix, sous le titre « Evaluation du programme « Ecole des Emotions » : développer les compétences socio-émotionnelles pour soutenir la réussite de l'enfant à l'école » reconnaît que ce projet répond aux exigences du comité d'éthique concernant la recherche en psychologie et en sciences de l'éducation.

Le comité d'éthique de l'UFR SPSE émet donc un avis favorable à la réalisation de ce projet.

Pour le comité d'éthique de l'UFR SPSE, ses coordonnateurs,

** The criteria used for the present evaluation are formalized in French in the "Guide of ethical evaluation of a research project" available on request from the Ethics Committee of the UFR SPSE*

The Ethics Committee of the UFR SPSE (Psychological Science and Education Science Formation and Research Unit) of University Paris Nanterre, upon reviewing the research project *Evaluation du programme "Ecole des émotions": developer les compétences socio-émotionnelles pour soutenir la réussite de l'enfant à l'école* , presented to them by Thomas Villemonteix, recognizes that this project meets the requirements of the Ethics Committee* concerning research in psychology and education sciences.

As a consequence, the UFR SPSE Ethics Committee expresses an opinion in favor of the realization of the present research project.

On behalf of the UFR SPSE Ethics Committee, the Coordinator

Régine Scelles — Gilles Séraphin

Attention, cet avis ne saurait se substituer à l'autorisation d'un Comité de Protection des Personnes (CPP) pour les recherches qui en relèvent, et ne dispense pas les promoteurs d'effectuer les démarches nécessaires concernant la protection de données (contact : dpo@liste.parisnanterre.fr)
